# Development and Validation of a Stability-Indicating RP-HPLC Method for the Determination of Process-Related Impurities and Degradation Products of Rabeprazole Sodium in Pharmaceutical Formulation

**DOI:** 10.3797/scipharm.1301-25

**Published:** 2013-03-17

**Authors:** Navneet Kumar, Dhanaraj Sangeetha

**Affiliations:** 1 Analytical Research and Development, Integrated Product Development, Dr. Reddy’s Laboratories Ltd., Bachupally, Hyderabad-500072, A.P, India.; 2 Department of chemistry, S.A.S., V.I.T. University, Vellore-632014, Tamilnadu, India.

**Keywords:** Development, Validation, Degradation, Stability-indicating, HPLC-UV, Rabeprazole, Impurity

## Abstract

The objective of the current study was to develop and validate a reversed-phase high-performance liquid chromatographic method for the quantitative determination of process-related impurities and degradation products of rabeprazole sodium in pharmaceutical formulation. Chromatographic separation was achieved on the Waters Symmetry Shield RP18 (250 mm × 4.6 mm) 5 μm column with a mobile phase containing a gradient mixture of solvent A (mixture of 0.025 M KH_2_PO_4_ buffer and 0.1% triethylamine in water, pH 6.4 and acetonitrile in the ratio of 90:10 v/v, respectively) and solvent B (mixture of acetonitrile and water in the ratio of 90:10 v/v, respectively). The mobile phase was delivered at a flow rate of 1.0 mL/min and with UV detection at 280 nm. Rabeprazole sodium was subjected to the stress conditions of oxidative, acid, base, hydrolytic, thermal, and photolytic degradation. Rabeprazole sodium was found to degrade significantly under acid hydrolysis, base hydrolysis, oxidative, and thermal degradation conditions. The degradation products were well-resolved from the main peak and its impurities, thus proving the stability-indicating power of the method. The mass balance was found to be in the range of 97.3–101.3% in all of the stressed conditions, thus proving the stability-indicating power of the method. The developed method was validated as per ICH guidelines with respect to specificity, linearity, limit of detection, limit of quantification, accuracy, precision, and robustness.

## Introduction

**Fig. 1:**
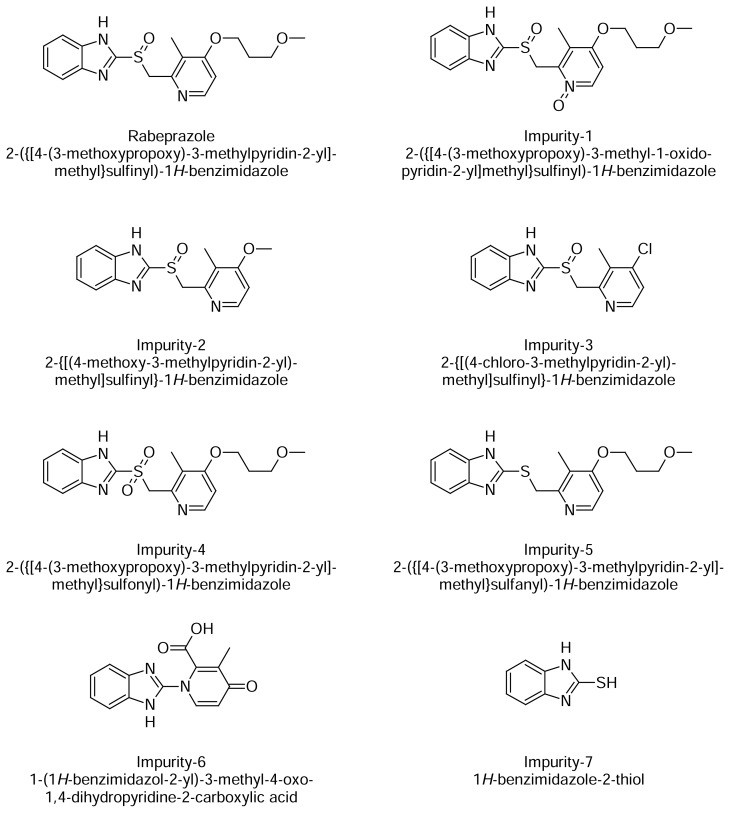
Chemical name and structures of Rabeprazole and its impurities.

The aim of the present study was to establish the inherent stability of rabeprazole sodium through stress studies under a variety of International Conference on Harmonization (ICH) recommended stress conditions. Rabeprazole sodium, 2-({[4-(3-methoxypropoxy)-3-methylpyridin-2-yl]methyl}sulfinyl)benzimidazole sodium salt (Figure 1), is a proton pump inhibitor which inhibits the action of H^+^–K^+^ ATPase in gastric parietal cells and is used for the treatment of peptic ulcers [1–3].

In the literature, there are few liquid chromatography (LC) methods previously reported for the determination of rabeprazole sodium in pharmaceutical preparation. Few liquid chromatography mass spectroscopy (LC-MS) methods were reported for the estimation of rabeprazole in biological fluids [4, 5]. The assay method [6–8] reported describes the quantification of rabeprazole sodium only, but it was out of scope because it did not separate and determine the impurities. A reversed-phase liquid chromatography (RP-LC) method is reported for the estimation of intermediates of rabeprazole sodium [9]. Also, the identification and characterization of new impurities and degradation products of rabeprazole sodium has been reported [10–14]. Rabeprazole sodium is not official in any major pharmacopoeia such as the United States Pharmacopoeia (USP), European Pharmacopoeia (EP), and British Pharmacopoeia (BP). Only one high-performance liquid chromatography (HPLC) method [15] is reported for the estimation of impurities present in the active pharmaceutical ingredient, rabeprazole sodium. The forced degradation study was not performed with a systematic approach in the above method. The objective of the stress testing is to anticipate the behavior of the drug product under the stability study. Forced degradation studies are essential to establish the stability-indicating power of the method. The reported paper claims that rabeprazole is stable under base hydrolysis and thermal stress conditions, while rabeprazole degrades significantly under these stress conditions. Subjecting the drug product samples to forced degradation is necessary to generate all possible degradation products that are used to demonstrate the specificity and selectivity of the method. Besides the reported known impurities in this method, we have observed two potential impurities during the forced degradation study of the drug product. Thus to the best of our present knowledge, no stability-indicating HPLC method has been reported for the estimation of all seven impurities of rabeprazole sodium in pharmaceutical formulation. Hence, we have developed a simple, reproducible stability-indicating reversed-phase HPLC method that can separate and determine the seven impurities of rabeprazole sodium, namely Imp-1, Imp-2, Imp-3, Imp-4, Imp-5, Imp-6, and Imp-7 (Figure 1). The developed LC method was validated with respect to specificity, limit of detection, limit of quantification, linearity, precision, accuracy, and robustness. Force degradation studies were performed on the placebo and drug products to show the stability-indicating nature of the method. These studies were performed in accordance with established International Conference for Harmonization (ICH) guidelines [16–18].

## Results and Discussion

### Development and Optimization of the Stability-Indicating Method

The main objective of the chromatographic method was to separate all known impurities and degradation products from each other and the rabeprazole peak formed under various stress conditions. The blend containing 500 μg/mL of rabeprazole sodium and 1.5 μg/mL of each of the seven impurities, prepared in diluent, was used for separation. All the impurities of rabeprazole sodium were subjected to separation by reversed-phase HPLC on a Waters Symmetry Shield RP18, 250 mm × 4.6 mm, 5 μm column with pH 3.0, 0.025 M potassium dihydrogen *ortho*-phosphate buffer as solvent A and water:acetonitrile in a 10:90 ratio as solvent B. The two compounds viz., rabeprazole sodium and Imp-3 were merged together and the peak tailing for rabeprazole was more than 2.0. To increase the resolution and reduce the peak tailing, solvent A was modified to a mixture of 0.025 M KH_2_PO_4_ buffer and 0.1% triethylamine in water, pH 6.4, and acetonitrile in the ratio of 90:10 v/v and the gradient program was optimized. The final chromatographic conditions are described under the “Chromatographic Conditions” section. Using the optimized conditions, all impurities and degradation products were well-separated from each other and rabeprazole and; the typical relative retention times for Imp-1, Imp-2, Imp-3, Imp-4, Imp-5, Imp-6, and Imp-7 were about 0.71, 0.85, 1.05, 1.12, 1.45, 0.18, and 0.53, respectively. The developed method was found to be specific for the determination for all seven impurities of rabeprazole sodium.

### Method Validation

The proposed method was validated as per ICH guidelines [17, 18]. The following validation characteristics were addressed: specificity, accuracy, precision, limit of detection and quantification, linearity, range, and robustness.

### System Suitability

System suitability was determined before sample analysis from a single injection of system suitability solution and duplicate injections of the standard solution containing 1.6 μg/mL rabeprazole sodium. The acceptance criteria were a USP tailing factor less than 2.0 and an area similarity ratio between 0.9 to 1.1 for the rabeprazole peak from duplicate injections of the standard and from the system suitability solution, where resolution should be a minimum of 1.5 between rabeprazole and Imp-3 peaks. All critical parameters tested met the acceptance criteria (Table 1).

### Specificity

**Fig. 2:**
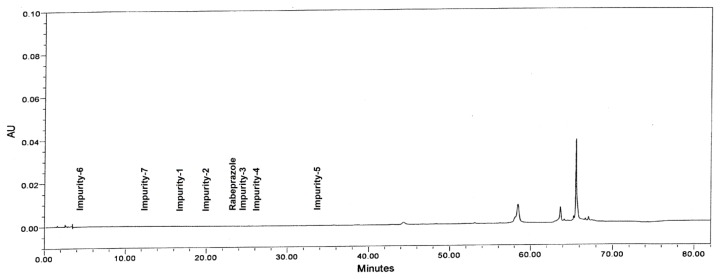
Typical chromatogram of the placebo.

Specificity is the ability of the method to measure the analyte response in the presence of its potential impurities and excipients. Placebo interference was evaluated by analyzing the placebo prepared as per test method. There was no interference due to the placebo and sample diluent at the retention time of rabeprazole and its impurities (Figure 2).

### Forced Degradation Studies

Forced degradation studies were performed at a 500 μg/mL concentration of rabeprazole sodium in tablet form to provide an indication of the stability-indicating property and specificity of the proposed method. All forced degradation samples were analyzed using a PDA detector to ensure the homogeneity and purity of the rabeprazole peak. All known impurities and unknown degradation products were well-separated under all of the forced degradation conditions employed, and the purity angle was found to be less than the purity threshold for the rabeprazole peak. Apart from the peaks’ homogeneity, the PDA spectrum for all of the related impurities and rabeprazole were compared against their standard spectrums. Identification of the impurities and rabeprazole was performed by comparing their PDA spectrums, purity plots, and their relative retention times (RRT) along with those of the standard and were found to be matching. The mass balance (%assay + % sum of all degradants + % sum of all impurities) results were calculated for all degradation samples and found to be more than 97.3% (Table 2). All the solutions used in the forced degradation studies were prepared by dissolving the drug product in a small volume of stressing agents. After degradation, these solutions were diluted with diluent to yield the stated rabeprazole sodium concentration of about 500 μg/mL. Conditions employed for performing the stress studies and the degradation behavior were as follows [16–18]:

### Acid Degradation

**Fig. 3:**
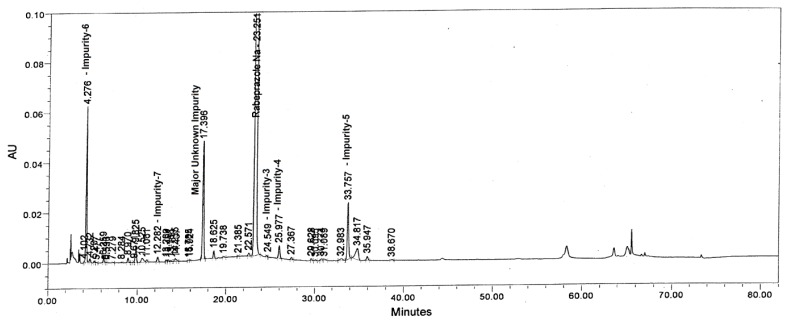
Typical chromatograms of Acid degradation sample

Tablet powder equivalent to 25 mg of rabeprazole sodium was transferred into a 50 mL volumetric flask, then 10 mL of diluent and 3 mL of 0.1 M HCl were added and mixed to dissolve the content completely. The flask was placed at 60°C in a water bath for 45 min. After 45 min, the flask was removed and placed on the benchtop to attain the laboratory temperature. To neutralize the sample, 3 mL of 0.1 M NaOH was added and made up to the volume with diluent and mixed well. The drug was found to be unstable under the aforementioned degradation conditions. The major impurity in the study was found to be Imp-5 (1.23%) with 2.06% as the maximum unknown degradant at an RRT of about 0.75 and total impurities of about 6.52% (Figure 3).

### Base Degradation

**Fig. 4:**
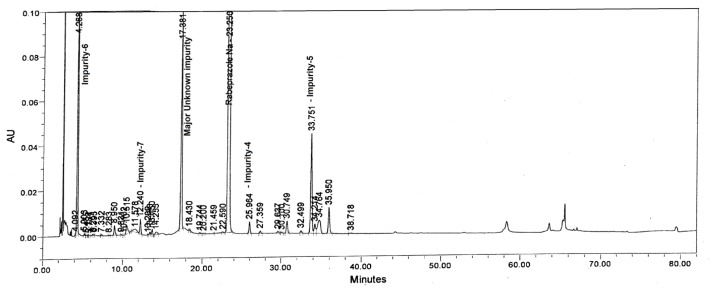
Typical chromatograms of Base degradation sample

Tablet powder equivalent to 25 mg of rabeprazole sodium was transferred into a 50 mL volumetric flask, then 10 mL of diluent and 5 mL of 0.5 M NaOH were added and mixed to dissolve the content completely. The flask was placed at 60°C in a water bath for 2 h. After 2 h, the flask was removed and placed on the benchtop to attain the laboratory temperature. To neutralize the sample, 5 mL of 0.5 M HCl was added and made up to the volume with diluent and mixed well. The drug was found to be very unstable under these stress conditions. The major degradants in the study were found to be Imp-5 (2.41%) with the maximum unknown degradant (4.61%) at an RRT of about 0.75 and total impurities of about 12.01% (Figure 4).

### Water Degradation

**Fig. 5:**
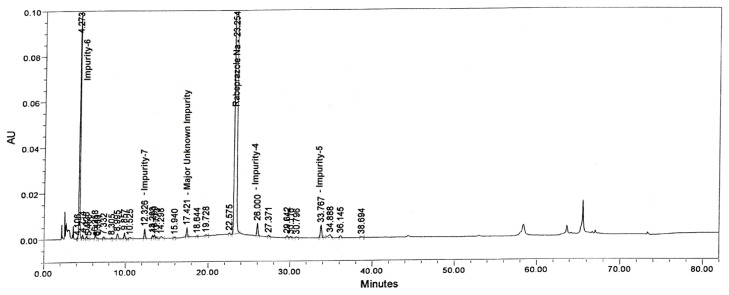
Typical chromatograms of Water degradation sample

Tablet powder equivalent to 25 mg of rabeprazole sodium was transferred into a 50 mL volumetric flask, then 10 mL of diluent and 10 mL of water were added and mixed to dissolve the content completely. The flask was placed at 60°C in a water bath for 3 h. After 3 h, the flask was removed and placed on the benchtop to attain the laboratory temperature and made up to the volume with diluent and mixed well. The drug degraded significantly under hydrolytic conditions. The major degradants in the study were found to be Imp-6 (2.01%) and an unknown degradant (0.27%) at an RRT of about 0.75 with total impurities of about 4.07% (Figure 5).

### Oxidation Degradation

**Fig. 6:**
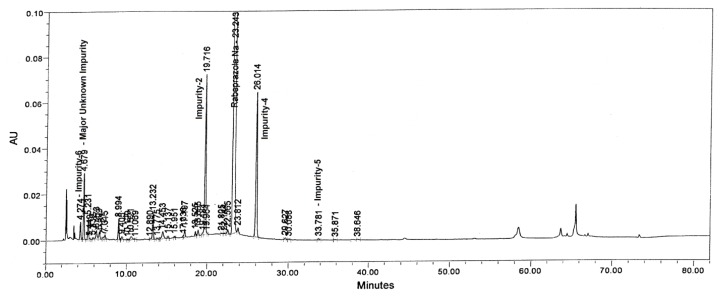
Typical chromatograms of Oxidative degradation sample

Tablet powder equivalent to 25 mg of rabeprazole sodium was transferred into a 50 mL volumetric flask, then 10 mL of diluent and 3 mL of 1% hydrogen peroxide were added and mixed to dissolve the content completely. The flask was placed at laboratory temperature for 30 min. After 30 min, the flask was made up to the volume with diluent and mixed well. The drug was found to be more labile to oxidative stress conditions. The major impurity in the study was found to be Imp-4 (3.27%) with 1.07% as the maximum unknown degradant at an RRT of about 0.20 and total impurities of about 8.50% (Figure 6).

### Thermal Degradation

**Fig. 7:**
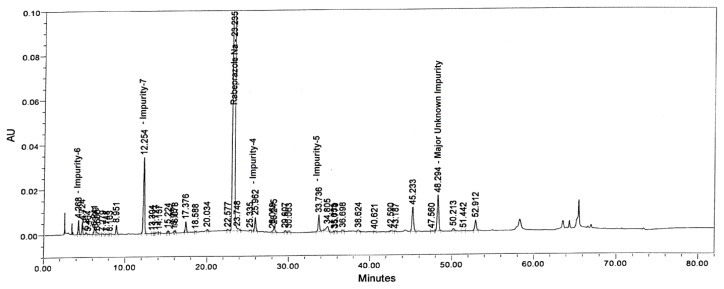
Typical chromatograms of Thermal degradation sample

To study the effects of temperature, an equivalent to 25 mg of rabeprazole sodium tablet powder was stored in a hot air oven at 105°C for 18 h. After 18 h, the sample was removed and placed on the benchtop to attain the laboratory temperature, dissolved in 35 mL of diluent, and diluted to 50 mL with diluent. Significant degradation was observed under the thermal stress studies. The major degradants in the study were found to be Imp-7 (0.52%) and an unknown degradant (1.63%) at an RRT of about 2.08 with total impurities of about 5.33% (Figure 7).

### Humidity Degradation

A saturated solution of potassium sulfate was prepared and placed in a dry glass desiccator at 25°C which produced about 85–90% of relative humidity. To obtain the effect of humidity on rabeprazole, a volumetric flask containing the sample (tablet powder equivalent to 25 mg of rabeprazole sodium) was kept in the aforementioned glass desiccator at 25°C/90% RH, and the sample was analyzed after seven days as described earlier under the “Test Preparation” section. The rabeprazole sample was quite stable under the humid conditions that were employed during the study. The sample showed no major degradation under the humidity conditions.

### Photolytic Degradation

Susceptibility of the drug product to light was studied [17]. Rabeprazole sodium delayed release tablets for photostability testing were placed in a photostability chamber and exposed to a white florescent lamp with an overall illumination of 1.2 million lux hours and near UV radiation with an overall illumination of 200 watt/m^2^/h at 25°C. Following the removal from the photostability chamber, the sample was prepared for analysis as previously described under the “Sample Preparation” section. Rabperazole was found to be highly stable under light exposure. No major degradant was observed in the sample exposed to both UV and visible light.

### Precision

The precision of the method was verified by repeatability and intermediate precision. Repeatability was checked by injecting six individual preparations of rabeprazole sodium samples spiked with its seven impurities (0.2% of each impurity with respect to 500 μg/mL rabeprazole sodium). The intermediate precision of the method was also evaluated using different analysts and different instruments and performing the analysis on different days. The % RSD for the area of Imp-1, Imp-2, Imp-3, Imp-4, Imp-5, Imp-6, and Imp-7 in the repeatability study was within 4.7% and during the intermediate precision study was within 4.1%, confirming good precision of the method. The % RSD values are presented in Table 3.

### Limits of Detection and Quantification

The LOD and LOQ for all impurities were determined at a signal-to-noise ratio of 3:1 and 10:1, respectively, by injecting a series of dilute solutions with known concentrations. The precision study was also carried out at the LOQ level by injecting six individual preparations and calculating the %RSD of the area for each analyte. The limit of detection, limit of quantification, and precision at the LOQ values for all seven impurities of rabeprazole sodium are reported in Table 3.

### Linearity

Linearity test solutions were prepared by diluting impurity stock solutions to the required concentrations. The solutions were prepared at six concentration levels from the LOQ to 200% of the specification level (ie. LOQ, 0.25, 0.50, 1.00, 1.50, and 2.00 μg/mL). The calibration curves were plotted between the responses of the peaks versus the analyte concentrations. The correlation coefficient obtained was greater than 0.998 (Table 3). The above result shows that an excellent correlation existed between the peak area and the concentration of Imp-1, Imp-2, Imp-3, Imp-4, Imp-5, Imp-6, and Imp-7.

### Accuracy

**Fig. 8:**
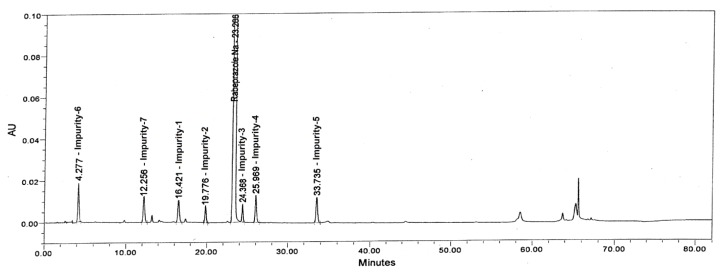
Typical chromatogram of Rabeprazole sodium sample spiked with its seven impurities

To determine the accuracy of the method, recovery experiments were conducted on the real sample by spiking the impurity blend solution. The study was carried out in triplicate using four concentration levels at the LOQ, 0.50, 1.00, and 1.50 μg/mL. The percentage recovery of impurities in the rabeprazole sample varied from 92.0 to 109.1%. The LC chromatogram of the spiked sample at the 0.2% level for all seven impurities in the rabeprazole sodium tablet sample is shown in Figure 8. The mean % recovery value of each impurity was obtained in the range of 92.0–109.1% which proves that the method is accurate. The % recovery values for the rabeprazole impurities are presented in Table 4.

### Robustness

To determine the robustness of the developed method, experimental conditions were deliberately altered and the resolution between rabeprazole and Imp-3, and system suitability parameters for the rabeprazole sodium standard were recorded. The variables evaluated in the study were the pH of the mobile phase buffer (± 0.2), column temperature (± 5°C), flow rate (± 0.2 mL/min), and % organic in the mobile phase (± 10%). In all of the deliberately varied chromatographic conditions, all analytes were adequately resolved and the elution order remained unchanged. The resolution between the critical pair of rabeprazole and Imp-3 was greater than 2.0 and the tailing factor for the rabeprazole peak from standard solution was less than 1.0 and the rabeprazole peak area ratio was within 0.9 to 1.1 (Table 5).

### Sample and Standard Solution Stability

The stability of rabeprazole and its impurities in solution was determined by leaving the test solutions of the sample and working standard in tightly capped volumetric flasks at room temperature for 48 h and measuring the amount of the seven impurities at 24 h intervals for 48 h. The variability in the estimation of all seven rabeprazole impurities was within ± 10% during the solution stability experiment. The results from the solution stability experiment confirmed that the standard solution and sample solutions were stable up to 48 h and 24 h, respectively.

## Experimental

### Chemicals and Reagents

The certified rabeprazole sodium working standard, tablets, and its impurities, namely Imp-1, Imp-2, Imp-3, Imp-4, Imp-5, Imp-6, and Imp-7 were supplied by Dr. Reddy’s Laboratories Limited, Hyderabad, India. The HPLC grade acetonitrile, analytical grade KH_2_PO_4_, triethylamine, and *ortho*-phosphoric acid were purchased from Merck, Mumbai, India. High-purity water was prepared by using the Milli-Q Plus water purification system (Millipore, Milforde, MA, USA).

### Instrumentation

The chromatography analysis was performed using the Waters Alliance 2695 separation module (Waters Corporation, Milford, USA) equipped with a 2489 UV/visible detector or a 2998 PDA detector (for the specificity and forced degradation studies), degasser, quaternary pump, and an autosampler system. The output signals were monitored and processed using Empower 2 software. A Cintex digital water bath was used for the hydrolysis studies. Photostability studies were carried out in a photostability chamber (Sanyo, Leicestershire, UK). Thermal stability studies were performed in a dry air oven (Cintex, Mumbai, India). The pH of the solutions was measured by a pH meter (Mettler-Toledo, Switzerland).

### Chromatographic Conditions

The method was developed using the Waters Symmetry Shield RP18 (250 mm × 4.6 mm) 5 μm particle size column with the mobile phase containing a gradient mixture of solvent A (mixture of buffer and acetonitrile in the ratio of 90:10 v/v, respectively) and solvent B (mixture of acetonitrile and water in the ratio of 90:10 v/v, respectively). The buffer contained a solution of 0.025 M potassium dihydrogen *ortho*-phosphate and 0.1% triethylamine in water, pH-adjusted to 6.4 with *ortho*-phosphoric acid. The gradient program (T(min)/%B) was set as 0/5, 50/65, 70/65, 72/5, and 82/5. The flow rate of the mobile phase was set at 1.0 mL/min. The column temperature was maintained at 25°C and the eluted compounds were monitored at the wavelength of 280 nm. The sample injection volume was 20 μL.

### Liquid Chromatography-Mass Spectrometry (LC-MS) Conditions

An LC-MS/MS system (Agilent 1100 Series liquid chromatograph coupled with the Applied Biosystem 4000 Q Trap triple quadruple mass spectrophotometer with Analyst 1.4 software, MDS SCIEX, USA) was used for the confirmation of the atomic mass number of the degradation compounds formed during the forced degradation studies. A YMC Pack C18, 150 mm × 4.6 mm, 5 μm column was used as the stationary phase. A 0.01 M ammonium acetate buffer and acetonitrile in the ratio of 95:5 v/v were used as solvent A and a 0.01 M ammonium acetate buffer and methanol in the ratio of 15:85 v/v were used as solvent B at a flow rate of 1.0 mL/min. The gradient program (T(min)/% solvent B) was set as 0/20, 40/80, 45/20, and 60/20. The analysis was performed in positive electrospray/positive ionization mode. The source voltage was 5000 V and the source temperature was 450°C. GS1 and GS2 were optimized to 30 and 35 psi, respectively. The curtain gas flow was 20 psi.

### Preparation of Standard Solution

Diluent was prepared by mixing methanol, Milli-Q water and diethylamine in the ratio of 80:20:0.1 v/v/v, respectively. A stock solution of rabeprazole sodium (0.4 mg/mL) was prepared by dissolving an appropriate amount of drug in the diluent. A working solution of 1.6 μg/mL was prepared from the above stock solution for the determination of related substances.

### Preparation of System Suitability Solution

A mixture of rabeprazole sodium (530 μg/mL) and all seven impurities (each 1.5 μg/mL) was prepared by dissolving an appropriate amount in diluent.

### Preparation of Sample Solution

Tablet powder equivalent to 25 mg rabeprazole sodium was dissolved in diluent with sonication for 30 min and diluted to give a solution containing 500 μg/mL of the drug. This solution was centrifuged at 4000 rpm for 10 min and filtered through 0.45 μm nylon membrane filter.

## Conclusions

The rapid gradient RP-HPLC method developed for the quantitative analysis of related substances of rabeprazole sodium in pharmaceutical dosage form is precise, accurate, linear, robust, and specific. Satisfactory results were obtained from the validation of the method. The method is stability-indicating and can be used for the routine analysis of production samples and to check the stability of the rabeprazole sodium tablets.

## Figures and Tables

**Tab. 1:** System suitability test results

Parameters	Specification	Observed values	

		Precision	Intermediate Precision
		
Resolution^a^	≥1.5	4.2	4.2
Standard area ratio	≥0.9 and ≤1.1	1.0	1.0
USP Tailing	≤2.0	1.0	1.0

a Resolution between Rabeprazole and Imp-3.

**Tab. 2:** Summary of forced degradation results

Stress Condition	% Impurity								% Degradation	Mass balance (%)

	I-1	I-2	I-3	I-4	I-5	I-6	I-7	MUSI^a^		
Acid hydrolysis	ND	0.02	0.02	0.27	1.23	0.70	0.03	2.06	6.52	98.5
Base hydrolysis	ND	0.02	ND	0.27	2.41	2.17	0.09	4.61	12.01	100.9
Oxidation degradation	ND	2.48	ND	3.27	0.04	0.11	ND	1.07	8.50	97.3
Thermal Degradation	ND	0.02	ND	0.31	0.41	0.09	0.52	1.63	5.33	101.3
Water Degradation	ND	ND	ND	0.28	0.29	2.01	0.07	0.27	4.07	101.0
Photolytic degradation	ND	ND	ND	0.20	ND	ND	ND	0.03	0.30	99.8
Humidity Degradation	ND	ND	ND	0.18	ND	ND	ND	0.04	0.29	100.4

a MUSI = Maximum un-specified impurity; ND = Not detected.

**Tab. 3:** Linearity and precision data

Parameter	Imp-1	Imp-2	Imp-3	Imp-4	Imp-5	Imp-6	Imp-7
LOD (μg/mL)	0.029	0.028	0.032	0.061	0.058	0.026	0.025
LOQ (μg/mL)	0.087	0.083	0.097	0.181	0.175	0.079	0.076
Correlation coefficient	0.999	0.999	0.999	0.999	0.999	0.999	0.999
Intercept	15.23	−357.57	−114.90	−962.70	1021.47	981.50	748.25
Slope	67617.6	59805.4	58174.2	43992.5	49474.1	123519.4	160103.1
Bias at 100% response	0.2%	1.3%	0.4%	1.5%	0.9%	1.8%	0.9%
Precision (%RSD)	1.2	2.4	3.6	1.1	0.6	1.8	2.3
Intermediate precision (%RSD)	2.0	4.1	3.1	3.4	2.1	1.3	1.6
Precision at LOQ (%RSD)	3.1	5.0	6.0	3.9	4.2	3.9	2.8

**Tab. 4:** Evaluation of accuracy study

Spiked Level^a^	% Recovery^b^						

	Imp-1	Imp-2	Imp-3	Imp-4	Imp-5	Imp-6	Imp-7
LOQ	94.2 ± 3.6	99.1 ± 2.6	95.7 ± 3.5	104.8 ± 0.4	104.7 ± 2.7	105.4 ± 2.0	96.5 ± 3.1
50%	96.0 ± 1.6	109.1 ± 3.3	95.5 ± 3.9	93.2 ± 2.7	106.1 ± 1.9	95.2 ± 3.2	103.2 ± 1.3
100%	96.8 ± 1.1	94.1 ± 3.0	98.9 ± 2.9	93.8 ± 3.3	95.8 ± 1.9	99.1 ± 1.9	101.8 ± 1.1
150%	92.0 ± 1.7	94.6 ± 1.3	93.8 ± 3.1	94.0 ± 2.8	103.3 ± 0.2	101.2 ± 1.9	98.5 ± 2.8

a Amount of seven impurities spiked with respect to 0.2% specification level individually;

b Mean ± %RSD for three determinations.

**Tab. 5:** Robustness results of HPLC method

Variation in chromatographic condition	Observed system suitability parameters		

	Standard area ratio ≥0.9 and ≤1.1	USP Tailing ≤2.0	Resolution a ≥1.5
Column Temperature 20°C	1.0	1.0	4.3
Column Temperature 30°C	1.0	1.0	3.0
Flow rate	1.0	1.0	4.4
0.8 mL/min			
Flow rate	1.0	1.0	3.1
1.2 mL/min			
Acetonitrile 90%	1.0	1.0	3.6
Acetonitrile 110%	1.0	1.0	3.6
Mobile Phase Buffer pH 6.2	1.0	1.0	4.4
Mobile Phase Buffer pH 6.6	1.0	1.0	4.3

a Resolution between Rabeprazole and Imp-3.
